# Ultrasound‐Guided Hip Pericapsular, Rectus Femoris Peritendinous, and Iliopsoas Bursa Injections Improve Extra‐articular Post‐arthroscopy Hip Pain

**DOI:** 10.1002/ars2.70042

**Published:** 2026-06-05

**Authors:** Meghan E. Sahr, Hanley Ong, Karthik Krishnan, Shu‐Han Wang, Danyal H. Nawabi, Theodore T. Miller

**Affiliations:** ^1^ Department of Radiology and Imaging Hospital for Special Surgery New York New York U.S.A.; ^2^ Department of Radiology Weill Cornell Medicine New York New York U.S.A.; ^3^ New York Presbyterian Hospital New York New York U.S.A.; ^4^ Epidemiology & Biostatistics Hospital for Special Surgery New York New York U.S.A.; ^5^ Sports Medicine Institute Hospital for Special Surgery New York New York U.S.A.

## Abstract

**Purpose:**

To determine the short‐ and medium‐term efficacy of ultrasound‐guided hip pericapsular, rectus femoris peritendinous, and iliopsoas bursa injections with anesthetic and corticosteroid to treat post‐arthroscopy hip pain, and if any operative variables correlated with response likelihood.

**Methods:**

Retrospective search for ultrasound‐guided injections performed from January 2015 to March 2024 was conducted. Inclusion criteria were history of hip arthroscopy to treat femoroacetabular impingement syndrome, persistent bothersome symptoms after conservative management including physical therapy, exclusion of intra‐articular pain sources; and treatment with simultaneous ultrasound‐guided injection of the hip capsule, rectus femoris tendon, and iliopsoas bursa. Responses immediately after the injection and at the next follow‐up visit were recorded. Likelihood of positive injection response was correlated with operative variables.

**Results:**

Fifty‐two injections in the same number of hips (28 men, 31 right hips) were performed a median of 308 days, range [121‐1300] following hip arthroscopy to treat femoroacetabular impingement syndrome. Immediately after the injection, 42 patients experienced improvement in symptoms, 2 had no improvement and 8 were unable to assess the immediate effect. The pre‐injection average visual analog score of 3.5 [SD ± 2.4] decreased to 0.4 [SD ± 1] immediately after the injections. At the next clinical follow‐up visit, 36 (69%) reported at least some improvement (5 (9%) mild, 13 (25%) moderate, and 18 (35%) marked). 14 (27%) patients reported no benefit and 2 (4%) were lost to follow‐up. At last clinical follow‐up, 29 cases had no or manageable symptoms, 7 underwent another surgery, 6 were discharged to pain management, and 10 had no further follow‐up data. There was no correlation between surgical variables and the degree of improvement at follow‐up (*P* = .266‐1).

**Conclusions:**

Patients with post‐arthroscopy hip pain with adequately treated intra‐articular factors may benefit from ultrasound‐guided injections targeting the joint capsule, rectus femoris origin, and iliopsoas bursa.

**Level of Evidence:**

Level IV, therapeutic retrospective case series.

## Graphical abstract

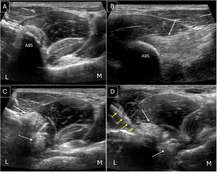

Hip arthroscopy performed for the treatment of femoroacetabular impingement syndrome (FAIS) has excellent outcomes.^1^, ^2^ A small subset of patients has a less satisfactory postoperative course with persistent pain, decreased range of motion, and function, for which an untreated or inadequately treated intra‐articular cause should be sought.^3^ Common indications for revision hip arthroscopy include residual cam deformity, labral tear, capsulolabral adhesions, dysplasia or microinstability, osteoarthritis, and capsular dehiscence.^4^ If intra‐articular pathology has been ruled out with physical examination, imaging, and possibly an intra‐articular injection, an extracapsular source should be considered.

Capsular disruption has been implicated in post‐arthroscopy pain and instability.^5^, ^6^, ^7^ All patients have postoperative capsular remodeling with increased thickness; capsular defects are associated with higher pain levels than those with healed capulotomies.^8^, ^9^, ^10^ In addition to capsular integrity, adhesions involving the capsule may also cause lingering symptoms, however the approach to their diagnosis remains poorly established.^11^ Although intra‐articular adhesions can be diagnosed with magnetic resonance (MR) arthrography, no physical examination maneuver or imaging modality can diagnose extra‐articular adhesions. Reddy et al. have performed pericapsular hydrodissection and corticosteroid injection under ultrasound in a cohort of 21 patients, with 12 experiencing at least partial pain relief, potentially in part to lysis of these extracapsular adhesions.^12^

Hip flexor tendinitis has also been implicated in post‐arthroscopy hip pain. Postulated causes include postoperative hypermobility or overactivation, adhesions or scarring, or improper physical therapy.^13^ Iliopsoas tendinitis has a reported postoperative incidence of 18% to 24%.^14^, ^15^ Of these, approximately half had resolution of symptoms with conservative management including physical therapy, non‐steroidal anti‐inflammatory drugs, and activity modification while the other half required iliopsoas bursa injection with corticosteroid. Rectus femoris is another hip flexor and anterior stabilizer which may also be damaged during arthroscopy. The reflected head is particularly vulnerable as it blends with the iliofemoral ligament and is in close proximity to instruments when anterosuperior acetabuloplasty or labral repair is performed.^16^

As extra‐articular causes of post‐arthroscopy hip pain are difficult to differentiate based on clinical and imaging examinations, patients with persistent symptoms after ruling out an intra‐articular cause and completing a course of physical therapy are referred for ultrasound‐guided injections around the hip capsule, the iliopsoas bursa and around the rectus femoris tendon simultaneously for diagnostic and therapeutic purposes. The purpose of this study was to determine the short‐ and medium‐term efficacy of ultrasound‐guided hip pericapsular, rectus femoris peritendinous, and iliopsoas bursa injections with anesthetic and corticosteroid to treat post‐arthroscopy hip pain, and if any operative variables correlated with response likelihood. We hypothesized that these injections would result in clinical improvement and that patients with larger capsulotomies would be more likely to benefit.

## METHODS

### Study Design and Patient Selection

The study was approved by the institutional review board (2024‐2290). We retrospectively identified patients treated for FAIS between January 2015 and March 2024. Inclusion criteria were patients who had undergone prior hip arthroscopy to treat FAIS by an experienced hip preservation surgeon (D.H.N.), had persistent bothersome symptoms after a workup including physical examination, imaging, and a trial of conservative management including physical therapy and non‐steroidal anti‐inflammatory drugs, and referral for simultaneous ultrasound‐guided injection of the rectus femoris origin, iliopsoas bursa, and hip capsule. Exclusion criteria were intra‐articular causes such as recurrent labral tear or non‐FAIS‐related extra‐articular causes revealed in the preceding workup, as well as other preceding hip surgeries, such as internal fixation of fracture. Patients without postinjection follow‐up documentation (immediate and first clinic follow‐up) or those with less than 1 year follow‐up period after the injections were excluded. If a patient had bilateral injections, the side performed last or the least symptomatic side (for simultaneous injections) was excluded to avoid correlated errors. The specific indications for the injections were most commonly persistent pain, followed by tightness, limited range of motion, and mechanical symptoms.

### Injection Technique

**FIGURE 1:**
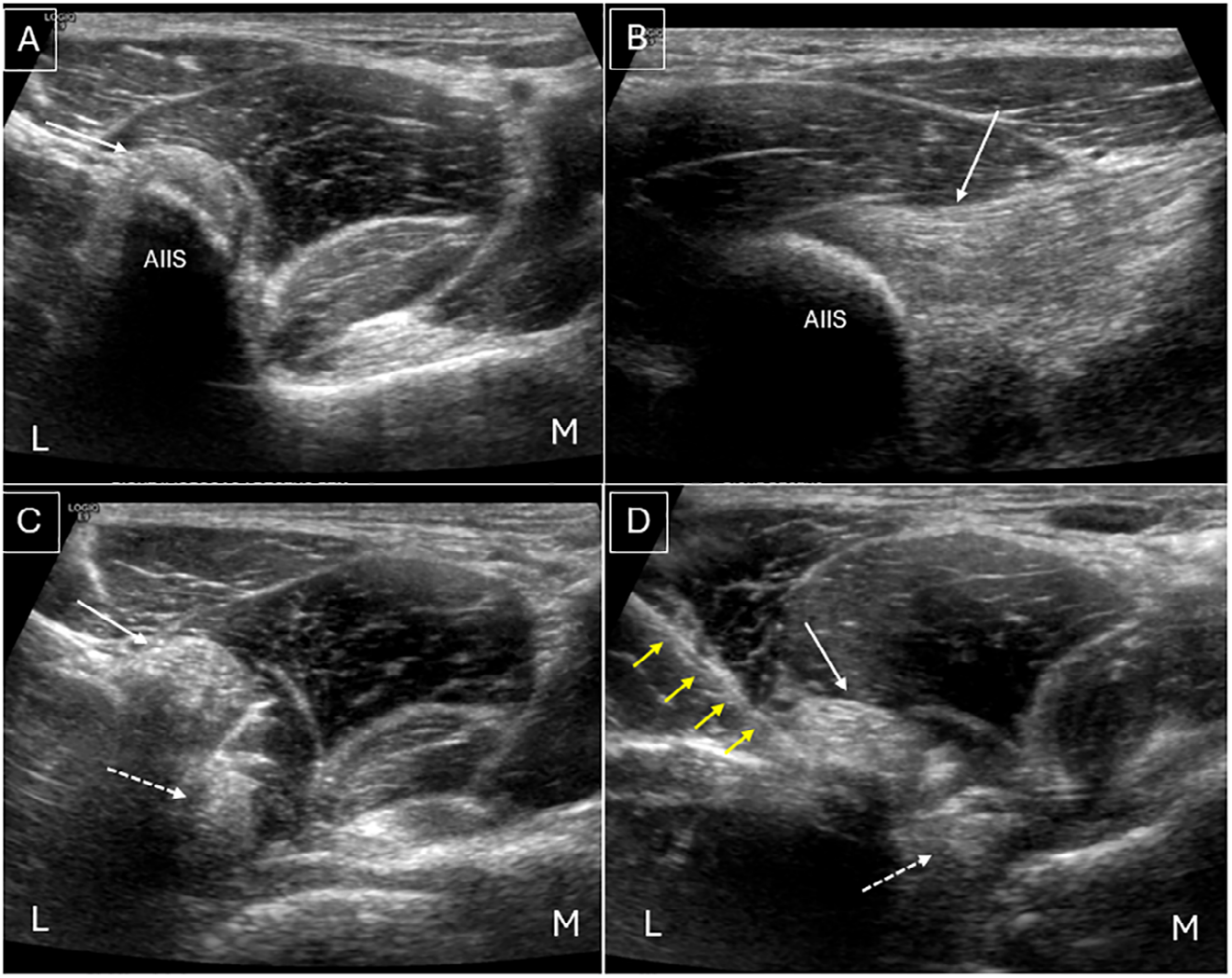
Rectus femoris peritendinous injection. Transverse (A) and longitudinal (B) ultrasound images of the origin of the direct head (solid arrow) in short and long axis, respectively. Slightly distal with the transducer in a transverse orientation, the direct (solid arrow) and indirect (dashed arrow) heads are visible in short axis (C). During injection at the level of figure part (C), the needle (yellow arrows) is advanced in plane to the transducer and positioned deep to both heads (white arrows) where the therapeutic steroid and anesthetic mixture is injected (D). (AIIS, anteroinferior iliac spine; L, lateral; M, medial.)

**FIGURE 2:**
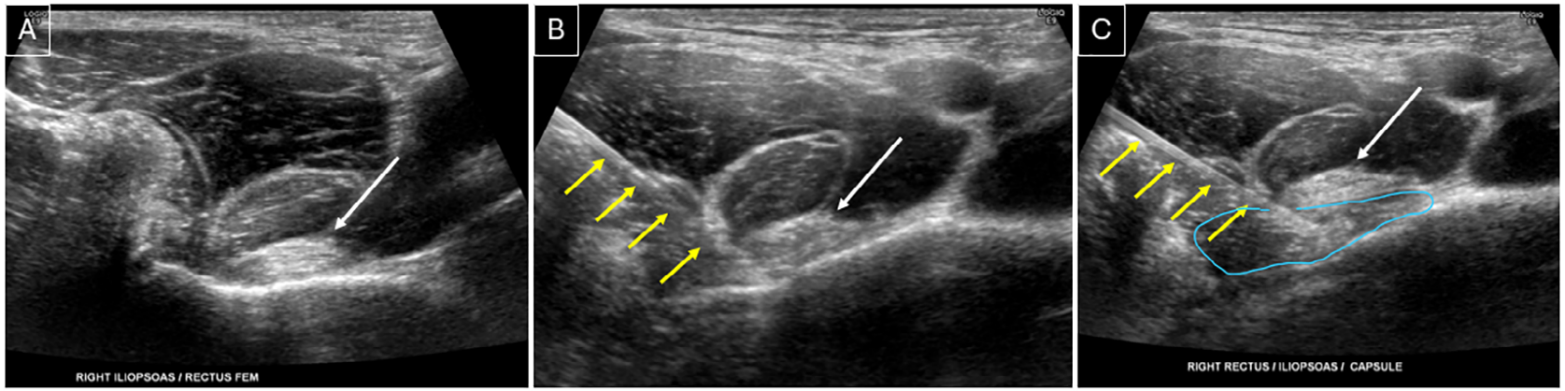
Iliopsoas bursa injection. Transverse ultrasound image of the iliopsoas tendon (solid white arrow) at the level of the pelvic brim (A). The needle (solid yellow arrows) is advanced in plane to the transducer until it is positioned on bone just lateral to the tendon within the potential space of the iliopsoas bursa (B). The therapeutic anesthetic and steroid mixture is then injected, distending the bursa (blue outline, C).

**FIGURE 3:**
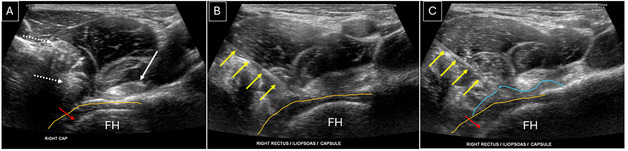
Hip pericapsular injection. Transverse ultrasound image of the proximal hip joint, just distal to the ilium (A). The iliopsoas (solid white arrow) and rectus femoris tendon (dashed white arrows) are also visible in short axis. During injection, the needle (yellow arrows) is advanced up to the hypoechoic joint capsule (orange outline) at the level of the femoral head (FH) at the level of the femoroplasty (red arrow) (B). The therapeutic anesthetic and steroid mixture (blue outline) is then injected, taking care to avoid intra‐articular injection (C).

Injections were performed by an experienced musculoskeletal radiologist (T.T.M.) using sterile technique under ultrasound guidance with a high‐resolution linear transducer (15 MHz), 1% lidocaine for local anesthetic, and a 22‐gauge, 3.5‐inch spinal needle to inject the therapeutic mixture. All injections were performed in a specialized procedure unit in the radiology department adapted for ultrasound‐guided procedures. Due to the proximity of the targeted structures, only one skin puncture was needed; the needle was simply partially retracted and redirected for each injection. The skin puncture site was typically located slightly lateral to the anteroinferior iliac spine. For rectus femoris, the direct head origin was identified with ultrasound in short axis at the origin off the anteroinferior iliac spine (Figure 1). The tendon was followed distally until the reflected head came into view. Utilizing a transverse approach, the needle was advanced until the tip was positioned deep to both heads, in‐plane with the transducer. A therapeutic mixture of 0.5 mL 1% lidocaine, 0.5 mL 0.25% bupivacaine and 1 mL triamcinolone 40 mg/mL) was injected. For the iliopsoas bursa, the tendon was localized at the level of the pelvic brim, just proximal to the hip joint, in short axis to the transducer (Figure 2). Using an in‐plane transverse approach, the same spinal needle was redirected into the bursa with the needle tip on the ilium just lateral to the tendon. A therapeutic mixture of 3 mL 1% lidocaine, 1 mL 0.25% bupivacaine, and 1 mL triamcinolone (40 mg/mL) was injected. For the hip capsule, the needle was again redirected distally to the superficial surface of the hip capsule at the level of the femoral head; over the femoroplasty if possible (Figure 3). The transducer was adjusted to show the needle in‐plane. A therapeutic mixture of 5 mL 1% lidocaine, 4 mL 0.25% bupivacaine, and 1 mL triamcinolone (40 mg/mL) was injected into the periarticular space. Care was taken to avoid intra‐articular injection.

### Data Collection

Our imaging report database (Montage Health Care Solutions, Philadelphia, PA) was retrospectively searched for ultrasound‐guided simultaneous injections of the iliopsoas bursa, anterior hip capsule, and rectus femoris origin performed by an experienced musculoskeletal radiologist (T.T.M.). The electronic medical record (Epic Systems, Verona, Wisconsin) for each patient was reviewed for the surgical details, including the arthroscopy date, hip laterality, if the procedure was primary or a revision, whether T‐capsulotomy, capsular repair or plication was performed, and the number of sutures used. Patient demographics of self‐reported sex, ethnicity and age at the time of injection were recorded. The time between arthroscopy and injection, the time between injection and the next clinical follow‐up interaction, and the injection response at both time points were recorded. Immediate response was assessed by the pre‐ and postinjection visual analog score score documented in the procedure note. Response at follow‐up was documented in the clinic note as a percentage or general category of improvement (none, mild, moderate, or marked). If a percentage was given, 0 was considered none, 1% to 33% mild, 34% to 66% moderate, and 67% to 100% marked. Further treatments such as ipsilateral intra‐articular injection, revision arthroscopy or other relevant subsequent surgeries were documented.

### Statistical Analysis

Descriptive statistics were used to summarize the characteristics of the study cohort. Continuous variables were reported as the median, minimum, and maximum values, as they are non‐normal distributions. Categorical variables were summarized using counts and percentages.

The primary outcome was the degree of improvement at follow‐up, categorized into two groups: “none” and “any improvement” (which combined mild, moderate, and marked responses). Independent variables included days from surgery to injection, T‐capsulotomy, capsular plication, number of sutures used in capsular closure, and operative intra‐articular biologic (BMAC/PRP) treatment. Continuous variables were compared between groups using the Wilcoxon rank‐sum test, while categorical variables were analyzed using Fisher's exact test. Statistical significance was defined as a *P* value < .05. All statistical analyses were performed using R version 4.3.2 (R Foundation for Statistical Computing, Vienna, Austria).

## RESULTS

**FIGURE 4:**
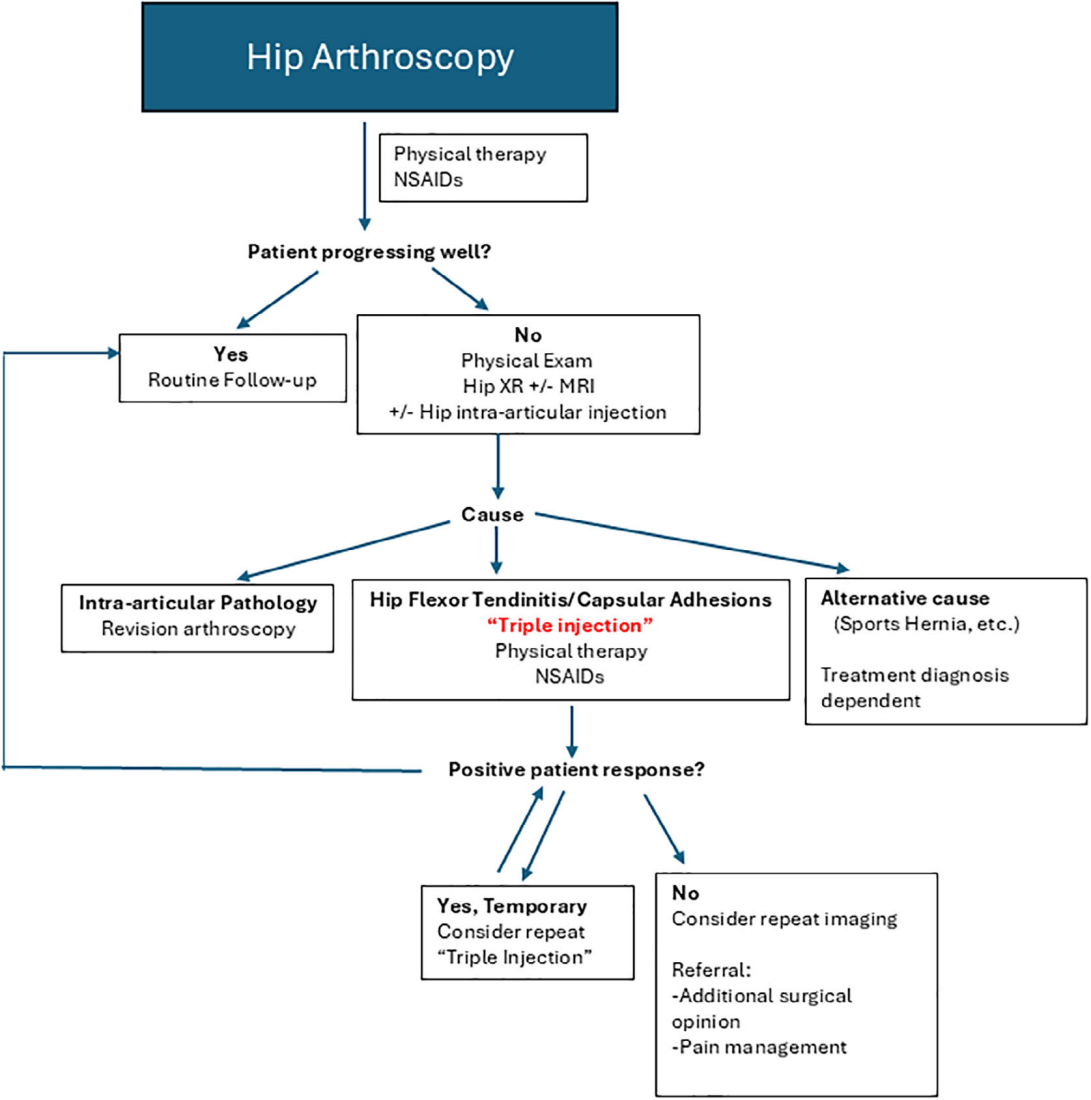
Flowchart depicting the post‐operative treatment algorithm for hip arthroscopy patients. (NSAIDs, non‐steroidal anti‐inflammatory drugs; MRI, magnetic resonance imaging.)

A single, experienced hip preservation surgeon (D.H.N.) performed 1100 hip arthroscopies to treat FAIS during the study period. All patients had undergone a routine postoperative rehabilitation program including physical therapy, but a small subset were either slow to progress or progressing poorly, as judged by the surgeon. Those patients with unrevealing evaluation were referred for the “triple injection.” The treatment algorithm is summarized in Figure 4. Most patients complained of postoperative pain (n = 49, 94%), while two complained of a sensation of “tightness” or limited range of motion, and one complained of mechanical symptoms (“catching”).

Fifty‐two hips (31 right, 21 left) in 52 patients had simultaneous triple injection of rectus femoris, the iliopsoas bursa, and hip capsule peritendinous injections with anesthetic and 40 mg of steroid at each site. The patient cohort consisted of 28 men and 24 women with an average age at injection of 33 years (range [16‐63]). Time from arthroscopy to injection averaged 309 days (range [121‐1300], SD 227). Forty‐nine surgeries were primary hip arthroscopies and 3 were revisions. All hips had the standard interportal capsulotomy, extended to the larger T‐capsulotomy in most cases (n = 35, 67%) Capsular closure was performed in all cases. Capsular plication was performed for most (n = 33, 63). Men were significantly more likely to have T‐capsulotomy and a larger number of sutures for closure. Categorical data is summarized in Table 1.

**TABLE 1:** Injection Group Categorical Data

	Combined	Male	Female	*P* Value (M v F)
Number of hips	52	28	24	‐
Age, average (yr)	32.8	33.9	32.3	.572
BMI (average [range])	24 [16.6‐33.5]	24.4	25.1	.502
Hip laterality (R:L), ratio	(31:21) 1.5	(16:13) 1.2	(16:8) 2	.416
Time scope to injection (days, median [range])	309 [121‐1300]	326 [162‐1300]	300 [121‐1010]	.389
Interportal capsulotomy	52 (100%)	28 (100%)	24	1.000
T‐capsulotomy (Y)	35 (67.3%)	24 (85.7%)	11 (45.8%)	**.003**
Number of sutures, average	7	4	6	**<.001**
Capsular plication (Y)	33:19 (63%)	19	14	.569
Intra‐articular biologics (Y)	16 (31%)	10	6	.549

*Note*: Statistically signifcant *P* values are highlighted in bold. BMI, body mass index; F, female; M, male; R:L, Right:Left; Y, yes; yr, year.

The majority of patients reported at least partial improvement in symptoms immediately following the injections due to the anesthetic effect (n = 42, 81%). Of those who had visual analog score documented in the procedure report, the average preinjection pain score improved from 3.5/10 before the injection to an average score of 0.4/10 after the injection. A minority were unable to assess immediate efficacy as their symptoms were related to activities that could not be reproduced in the procedure suite (n = 8, 14%). Only 2 patients reported no immediate improvement (4%).

Clinical follow‐up was obtained after 49 of the 52 injections. Three patients were lost to follow‐up and for one, injection response was not documented. The median time from injection to the first follow‐up visit was 43 days (range [4‐1167]). After this, follow‐up varied between groups, with responders discharged for follow‐up as needed and nonresponders receiving further imaging, injections, or referrals. There was no correlation between a positive injection response and operative variables (Table 2). There was a trend toward positive injection response in patients who did not have a T‐capsulotomy or receive intra‐articular biologics. There was no difference in the response likelihood in those who had capsular plication. The length of time between the surgery and injections for responders and nonresponders was not significantly different (mean 297 vs 397 days, *P* = .42).

**TABLE 2:** Operative Characteristics Versus Injection Response

			Improvement at Follow‐Up		*P* Value
			Y	N	
T‐capsulotomy	Y	All	22, 68.6%	11, 31.4%	.341 (all)
		M	14	10	.266 (M)
		F	8	1	.616 (F)
	N	All	14, 82.4%	3, 17.7%	
		M	4	0	
		F	10	3	
Capsular plication	Y	All	24, 72.7%	9, 27.3%	1 (all)
		M	13 M	6 M	.678 (M)
		F	9 F	3 F	.594 (F)
	N	All	14, 73.7%	5, 26.3%	
		M	5 M	4 M	
		F	9 F	1 F	
BMAC/PRP	Y	All	10, 62.5%	6, 37.5%	.316 (all)
		M	6 M	4 M	1 (M)
		F	4 F	2 F	.292 (F)
	N	All	28, 77.8%	8, 22.2%	
		M	12 M	6 M	
		F	14 F	2 F	

BMAC, bone marrow aspirate concentrate; F, female; M, Male; N, no; PRP, platelet‐rich plasma; Y, yes.

At the first clinical follow‐up encounter, 36 injections had resulted in at least partial symptomatic improvement (65% of original cohort). Of these, improvement was described as mild (5), moderate (13), or marked (18). Interestingly, the 2 patients who reported no immediate postinjection improvement were both positive responders at follow‐up. Seven of 8 patients who could not assess the immediate injection efficacy were improved at follow‐up. Of the responders, 25 had either absent or manageable symptoms and were discharged to follow‐up as needed and had no additional complaints during the 1 year period after the injections. Of the remaining 9 short‐term responders, 6 had either persistent or recurrent symptoms treated with either repeat triple injection (4) or intra‐articular hip injection (2) with mixed responses, one was referred to pain management, one underwent revision hip arthroscopy, and another was referred for capsular reconstruction.

In 13 cases, patients reported they were no better than before the injections at the first clinical follow‐up encounter (24% of original cohort). Of these, 4 were ultimately referred to pain management, 1 underwent intra‐articular hip injection with a positive response, 4 were referred for additional surgery (obturator hernia repair (1), adductor tendon repair (1), and revision hip arthroscopy (2), 3 improved with conservative management and 1 was lost to follow‐up.

## DISCUSSION

Almost all FAIS post‐arthroscopy patients experience temporary pain relief immediately after simultaneous triple injection of the rectus femoris tendon, iliopsoas bursa, and hip joint capsule due to the anesthetic, and most reported improvement at the next clinical follow‐up. No operative variables correlated with injection response, although there was a trend toward positive response in those who did not have a T‐capsulotomy or receive intra‐articular biologics at surgery. Our cohort had a relatively high rate of capsular plication although there was no correlation of this variable with injection response. These patients had a component of dysplasia or risk of microinstability that the surgeon was attempting to address and their underlying anatomy may have increased the risk of a less favorable course. There was no difference in likelihood of response between men and women. Men had a significantly higher rate of T‐capsulotomy and thus a higher number of sutures used for closure, as expected given the larger cam deformities usually seen in men.^17^

Despite the excellent published outcomes of hip arthroscopy for the treatment of labral tears and FAIS, some patients experience persistent or recurrent symptoms.^18^ There is growing interest in the treatment of extra‐articular causes of post‐arthroscopy hip pain and limited range of motion. Iliopsoas irritation, extra‐articular adhesions between the iliopsoas tendon, rectus femoris injury during acetabular rim debridement, and capsular deficiency or delayed healing are known causes.^18^, ^19^ Non‐invasive interventions to reduce the formation of adhesions include passive and active range of motion exercises, and pharmacological treatments such as losartan which blocks TGF‐β1‐induced fibrosis.^19^ Reddy et al.^12^ performed ultrasound‐guided periarticular hip hydrodissection and injection with corticosteroid with decreased postoperative pain in 12 of 21 patients. Our pericapsular injection was performed using a similar technique including the volume of injectate (10 mL in ours vs 10 to 12 mL in theirs). Efficacy is thought to result from a combination lysis of extra‐articular adhesions and the anti‐inflammatory effect of corticosteroid on the healing capsule. Notably, our triple injection intervention resulted in a positive response in a larger proportion of patients in a larger cohort, suggesting simultaneous treatment of hip flexor tendinitis and/or higher steroid doses are beneficial.

Capsular healing evaluated on magnetic resonance imaging (MRI) has been correlated with patient outcomes. Strickland et al.^20^ prospectively evaluated 30 hips in 15 patients who underwent simultaneous bilateral hip arthroscopy with one hip in each patient randomized to undergo interportal capsulotomy repair, with the other left unrepaired. At 6 weeks, MRI showed a non‐significant greater number of defects in the nonrepair group, with all capsules progressing to healing at 24 weeks after surgery, suggesting healing may progress more slowly without repair. In our study, patients who did not have T‐capsulotomy showed a non‐statistically significant trend toward a better injection response even though capsular repair was performed for all cases, counter to our expectations. This may be because cases with less capsular disruption heal more rapidly and potentially with less scarring. Patients therefore may have trended toward greater improvement whether the injections were performed or not. It remains unknown whether larger capsulotomies, such as T‐capsulotomies with closure heal more slowly than smaller capsulotomies with closure; there is no data that suggests larger capsulotomies result in worse outcomes.^21^ Data regarding post‐arthroscopy capsular thickness on MRI is mixed, with some investigators finding thinner capsules correlate with improvements in patient pain, function and return to sport^10^ and others finding no correlation.^8^ Notably, there was no correlation between the interval between arthroscopy and the “triple injection” and response; therefore, the injection can be considered an option for patients outside the immediate postoperative period.

Hip flexor tendinitis is also a known cause of extra‐articular hip pain following hip arthroscopy. In a retrospective analysis of 258 arthroscopic hip procedures for treatment of FAI and labral tear by Campbell et al.,^15^ 18.2% developed clinical symptoms of iliopsoas tendinitis. Those who did not respond to conservative management were referred for ultrasound‐guided cortisone injection into the iliopsoas bursa, with 18 of 24 improving after the injection by the 1‐year follow‐up visit. Ultrasound visualization of iliopsoas tendinosis or bursal fluid was encountered in all of their cases, which contrasts with our experience. Another retrospective study of 252 patients after hip arthroscopy by Adib et al.^14^ reported a 24% incidence of iliopsoas tendinitis diagnosed by physical examination. Similar to Campbell et al., about half responded to conservative measures with 32 requiring cortisone injection an average of 25 weeks after surgery. Enduring positive responses were produced in 25 patients while 7 had transient relief and were later successfully treated with tenotomy. Notably, our triple injections were performed later than in both studies, an average of 1 year after surgery; however, the response rates were similar. Neither study was able to correlate any preoperative characteristics or surgical variables with likelihood of developing iliopsoas tendinitis; patients in the injection cohort had lower patient reported outcome measures than the control group at 1 year after surgery.

There are few guidelines in the literature that describe an appropriate cortisone dosage for pain management injections in regard to efficacy.^22^ The recommended dose of triamcinolone acetonide for large joints is 40 mg per joint with a total of 80 mg at once, according to the manufacturer.^23^ Most of the literature provides short‐term results for large joint injections; however, our triple injection targets were periarticular. Doses of 40 to 80 mg have been shown to be efficacious for the greater trochanteric and iliopsoas bursae.^22^ Our triple injection dosing of 120 mg exceeds this, although the dose injected at each site is within the recommended range. Studies examining steroid efficacy and dose at other anatomic sites have mixed results, with some showing longer pain relief with higher doses and others showing no difference.^24^ It is unknown whether a lower cumulative steroid dose would have had similar efficacy in our patient cohort, although none reported unpleasant side effects following the injections. It is also unknown to what extent the hydrodissection effect contributes to any improvement.^12^

Adverse effects of corticosteroids include decreased bone mineral density, inhibition of the hypothalamic pituitary axis, increased blood sugar and infection.^22^ High corticosteroid doses around the hip raise concern for potential rapidly progressive osteoarthritis. Animal studies have shown in vitro and in vivo chondrotoxicity. Recent retrospective studies have raised concerns that intra‐articular corticosteroid injections may cause rapidly progressive osteoarthritis,^25^ however prospective evidence is mixed.^26^ Studies used to support intra‐articular cortisone injections were underpowered to detect rapidly progressive osteoarthritis, while those reporting an increased incidence following injection were subject to bias.^26^ It is unclear whether this is a relevant potential risk of the triple injection to our patient cohort without arthritis undergoing periarticular injections. The iliopsoas bursa communicates with the hip joint in approximately 15% of individuals, an occurrence that increases in pathologic states.^27^, ^28^ Some intra‐articular spread may therefore inadvertently occur even if ideal technique is employed. Use of the lowest effective steroid dose possible is therefore desired to mitigate against the potential risk of joint destruction.

Our study reports promising patient outcomes from the ultrasound‐guided triple injection technique in a subset of hip arthroscopy patients that are slow to improve. It is unknown whether equally favorable responses could be obtained from different injection combinations, such as the hip capsule and rectus femoris only, or with lower cortisone doses. Different surgeons at our institution order injections in different combinations, and their efficacy could be examined in further studies with a larger patient cohort. We were unable to identify patient or surgical factors that could predict who might benefit from triple injection. Prior studies have shown MRI can accurately depict adhesions and capsular defects, but whether they are predictive of injection response remains a subject for further study.^29^, ^30^

### Limitations

Limitations of our study include its retrospective design without a control group and the small sample size; therefore, correlation rather than causation is established. It was difficult to find fair controls as the patients in the injection subset had lower preinjection patient reported outcome measures (PROMs) than average, which is why they were referred for treatment. Aside from visual analog score scores, we had no additional patient reported outcome measures data with which to gauge efficacy. The injections were performed by a single radiologist for patients referred by a single surgeon; therefore, the results might not be generalizable to others with less ultrasound‐guided procedure experience or those using slightly different arthroscopic techniques.

## CONCLUSIONS

Patients with post‐arthroscopy hip pain and adequately treated intra‐articular factors may benefit from ultrasound‐guided injections targeting the joint capsule, rectus femoris origin, and iliopsoas bursa.

## DISCLOSURES

The authors (M.E.S., H.O., K.K., S‐H.W., D.H.N., T.T.M.) declare that they have no known competing financial interests or personal relationships that could have appeared to influence the work reported in this paper.
